# Strong effects of genetic and lifestyle factors on biomarker variation and use of personalized cutoffs

**DOI:** 10.1038/ncomms5684

**Published:** 2014-08-22

**Authors:** Stefan Enroth, Åsa Johansson, Sofia Bosdotter Enroth, Ulf Gyllensten

**Affiliations:** 1 Department of Immunology, Genetics, and Pathology, Biomedical Center, SciLifeLab Uppsala, Uppsala University, SE-75108 Uppsala, Sweden; 2 Uppsala Clinical Research Centre, Uppsala University, SE-75237 Uppsala, Sweden; 3 Department of Medical Sciences, Uppsala University, SE-75185 Uppsala, Sweden

## Abstract

Ideal biomarkers used for disease diagnosis should display deviating levels in affected individuals only and be robust to factors unrelated to the disease. Here we show the impact of genetic, clinical and lifestyle factors on circulating levels of 92 protein biomarkers for cancer and inflammation, using a population-based cohort of 1,005 individuals. For 75% of the biomarkers, the levels are significantly heritable and genome-wide association studies identifies 16 novel loci and replicate 2 previously known loci with strong effects on one or several of the biomarkers with *P*-values down to 4.4 × 10^−58^. Integrative analysis attributes as much as 56.3% of the observed variance to non-disease factors. We propose that information on the biomarker-specific profile of major genetic, clinical and lifestyle factors should be used to establish personalized clinical cutoffs, and that this would increase the sensitivity of using biomarkers for prediction of clinical end points.

## Abstract (web summary)

Protein biomarkers could play an important role in the diagnosis and management of diseases. Here the authors investigate the impact of genetic, clinical and lifestyle factors on 92 protein biomarkers for cancer and inflammation and suggest that personalized biomarker thresholds should be used in cancer management.

## Graphical abstract

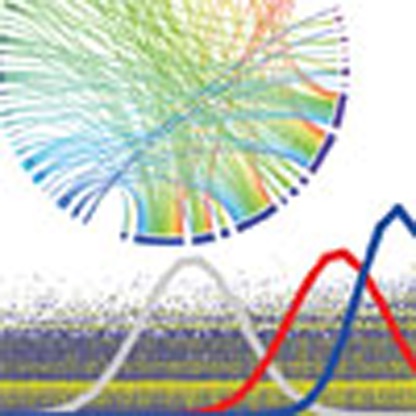

A number of protein biomarkers are used for diagnosis and management of cancers and other diseases. Examples include prostate-specific antigen^1^ used to screen for prostate cancer, the ovarian cancer-related tumour marker CA125 (ref. 2) and IL-6, which is a drug target in rheumatoid arthritis (RA)^3^. Ideal biomarkers for early diagnosis should be uniquely present, or overexpressed, in the malignant tumour or blood and not influenced by confounding factors. Most current biomarkers have a function in the normal cell, taking part in, for example, signalling pathways, controlling growth, apoptosis and/or inflammation^4^. These are not uniquely expressed in the malignant tissue and their expression level is affected by a number of factors, such as the individual’s genetic and physical constitution, lifestyle and medication. A detailed understanding of potential confounding factors and their effect size is therefore a necessary prerequisite in the evaluation of the rapidly growing number of candidate biomarkers^5^. The discovery of putative biomarkers for early identification and management of cancer has been greatly facilitated by high-throughput, genome-wide assays. Gene expression analyses have discovered numerous genes that are differentially expressed between malignant and benign tissues^6^, but few have proven suitable as biomarkers, mainly because the mRNA levels do not correlate well with protein abundance^7^. Large-scale studies of protein abundance, on the other hand, have been hampered by lack of high-throughput methods. High-resolution mass spectrometry can be used to examine the underlying genetic contribution to profiles of circulating proteins and effect of environmental covariates, but the resolution is limited by the peptide spectra used and detection sensitivity^8^. An alternative is to use antibody-based measurements, which targets individual, preselected, sets of proteins.

Here we aim to understand the factors that influence normal variation in plasma levels of established and potential biomarkers for cancer, autoimmune diseases and inflammation with the specific goal to facilitate the establishment of individualized clinical cutoffs. To this end, we use the highly sensitive and specific proximity extension assay (PEA)^9^ to estimate the abundance of 92 established or potential biomarkers in plasma from 1,005 individuals from a longitudinal cross-sectional population-based study in Sweden. The biomarkers we analyse here constitute a research panel directed against multiple cancers and also contain proteins implicated in autoimmune diseases such as RA and Graves’ disease. PEA combines two dedicated antibodies with a real-time quantitative PCR (qPCR) reaction to achieve high specificity and a wide dynamic range. This technology can be multiplexed without introducing crosstalk, while still maintaining its high specificity and sensitivity^10^. We first determine the effect of a wide range of clinical variables and lifestyle factors, including age, sex, blood pressure, blood group or body mass index (BMI), medication and smoking, on biomarker levels. Then we study the heritability of each biomarker, and by using high-resolution genetic single-nulecotide polymorphism (SNP) array data and whole-exome sequencing, we perform a genome-wide association study (GWAS) for each biomarker. This study is the first to measure biomarker abundance on a large scale using a single technology in a general population, in order to identify contributing factors to normal variation. To our knowledge, only one previous study has studied the genetic association of multiple proteins in the general population. Melzer *et al*.^11^ investigated 42 proteins using a variety of assays, prohibiting protein-to-protein comparisons, and also did not investigate the protein-specific profiles of covariates. Here by integration of genetic, clinical and lifestyle data, we identify the set of biomarker-specific factors that can be used to determine appropriate individual clinical cutoffs, and thereby enable a more efficient use of each biomarker in personalized cancer management.

## Results

### Biomarker measurements

**Figure 1:**
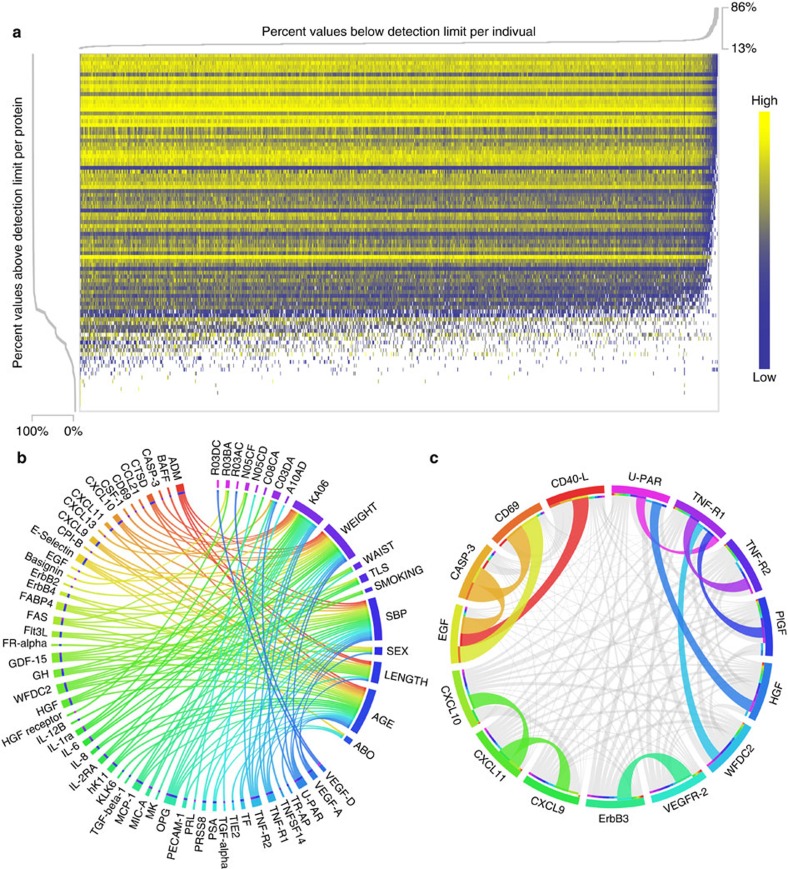
Characteristics of the PEA measurements. (**a**) Intensities of PEA values and proportion of proteins and individuals above detection limit. In the heatmap, individuals are in columns and proteins are in rows. Heatmap colours represent ddCq-values ranging from low (blue) to high (yellow) with measurements below detection limit coded white. (**b**) Significant covariates in relation to each protein. Covariates are listed from the upper right part of the circle (12 o'clock to 4) and connections illustrate significant (*P*-value <0.05, Bonferroni adjusted) contributions to PEA variance. (**c**) PEA to PEA correlations, coloured connections represent a correlation coefficient (*R*^2^) greater than 0.5. The width of the connection reflects the magnitude of the squared correlation coefficients. All correlations coefficients (*R*) were positive.

The abundance of 92 proteins (Supplementary Data 1), representing a panel of established and potential biomarkers for cancer and inflammation, were measured in blood plasma of 1,005 individuals from the Northern Sweden Population Health Study (NSPHS), using PEA and qPCR. A total of 77 of the proteins had levels above the detection limit in at least 80% of our samples, with 91.3% (70,651 of 77,385) of qPCR reactions being successful. In the remaining 15 proteins, 96.8% (14,598 of 15,075) of the protein levels were below the detection limit. Also, 96.5% (970 of 1,005) of our samples passed quality control on an individual level. The abundance and distribution of the normalized measurements (delta delta Cq (ddCq)-values) of all the proteins in all samples are illustrated in Fig. 1a, with estimates under the detection limits coloured white. Details on normalization and initial quality control are given below in the Methods section. The proteins with little or no measurable abundance in our samples were: stromelysin-1, GM-CSF (granulocyte-macrophage colony-stimulating factor), estrogen receptor, CA242 (cancer antigen 242), IL-2 (interleukin-2), epiregulin, betacellulin, IL-4, interferon-γ, IL-7, TNF (tumour necrosis factor), CEA (carcinoembryonic antigen-related cell adhesion molecule 5), MYD88 (myeloid differentiation primary response protein MyD88), mucin-16 and regenerating islet-derived protein 4. It cannot be ruled out that storage-time and protein degradation could be an influencing factor for these 15 proteins, and previous studies have quantified this specifically for CEA^12^.

### Epidemiological associations

To study the effect of clinical and lifestyle factors, we selected 158 phenotypic covariates, including age, sex, blood pressure, BMI, tobacco use, medication, lifestyle (occupation) and sample collection round (2006 or 2009) from the comprehensive set of clinical data available for NSPHS. A multiple linear regression model showed a total of 18 phenotypic covariates to have a significant effect (*P*-value<0.05, Bonferroni adjusted) on one or more of 52 of the 77 proteins (Table 1). Factors such as age or weight influenced a broad range of proteins, whereas medication affected specific proteins (Fig. 1b). Notably, smoking affected two proteins, WFDC2 (WAP four-disulfide core domain protein 2) and IL-12, whereas the traditional Swedish moist tobacco product, ‘snus’ did not have any significant effects, in line with a previous study on effects of tobacco use on DNA methylation^13^. We also found large effects (nominal *P*-value ranging from 1.8 × 10^−4^ to 2.3 × 10^−7)^ of ABO blood group on three proteins; E-selectin, PECAM-1 (platelet endothelial cell adhesion molecule) and TIE2 (angiopoietin-1 receptor). The connection between E-selectin and blood groups is known^14^,^15^, but the effect on PECAM-1 and TIE2 has not been described previously. The medication in NHPHS had been investigated using a questionnaire and the reported medications were annotated using the Anatomical Therapeutic Chemical (ATC) classification system. Among the commonly used medications, dihydropyridine derivatives (ATC: C08CA, 54 users), often used to treat hypertension, were correlated to increased IL-6 levels, whereas glucocorticoids (ATC: R03BA, 26 users) lowered both Basigin and hepatocyte growth factor (HGF) receptor levels. Apart from C08CA, no other hypertensive treatment was correlated with high IL-6 levels. Interestingly, the usage of selective β-2-adrenoreceptor agonist (ATC: R03AC, 13 users), which is commonly found in asthma inhalators, decreased the level of circulating vascular endothelial growth factor D (VEGF-D), which is implicated in the metastasis of non-small lung cancer^16^,^17^. A detailed description of all investigated covariates and their association with protein levels is given in Supplementary Data 2. The largest fraction of variance explained by a single clinical or environmental covariate was age, which accounted for 27% of the variation seen for WFDC2. The influence on WFDC2 of age and smoking has previously been reported^18^, but we found that the fraction of variance explained by smoking in our data to be only 1.7%, which is much less than for systolic blood pressure (14.3%) or loop-diuretics (ATC: C03CA, plain sulfonamides, 7.2%). However, these covariates are not necessarily independent as blood pressure and use of medication is related to age.

### Correlations between biomarkers

Inter-biomarker correlation was investigated using abundance levels adjusted for significant clinical and lifestyle covariates. These was then rank-transformed into normally distributed values and used to identify 12 pairs with a Spearman’s Rho *R*^2^ greater than 0.5 (Fig. 1c). The highest correlation was found between CASP-3 (caspase-3) and CD69 (early activation antigen CD69; *R*^2^=0.85). CASP-3 was also highly correlated with epidermal growth factor (*R*^2^=0.81), which in turn was highly correlated with CD69 (*R*^2^=0.78). The strong correlation between some of the biomarkers does not appear to be reflected at the transcription levels. For instance, the Illumina Body Map^19^,^20^,^21^ suggests that CD69 and caspase-3 both are expressed in leukocytes, lymph nodes and adrenal glands (for example, 3 of 16 investigated tissues). In data from leukocytes of 80 controls^22^, there was only a weak correlation between the expression levels of CD69 and CASP-3 (*R*^2^=0.13), suggesting that the high correlation observed at the protein level is either because of post transcriptional regulation, for example, epigenetic regulation, or owing to expression patterns in distinct cell types. Several of the 12 pairs that were highly correlated were proteins with similar functions, such as C-X-C motif chemokine (CXCL)-9, -10, -11, and TNF-R1 and TNF-R2, whereas in other cases apparently unrelated proteins were highly correlated. These correlations may reflect as yet unknown patterns of co-regulation, and bring into question their value as independent biomarkers.

### Heritability and genetic association

**Figure 2:**
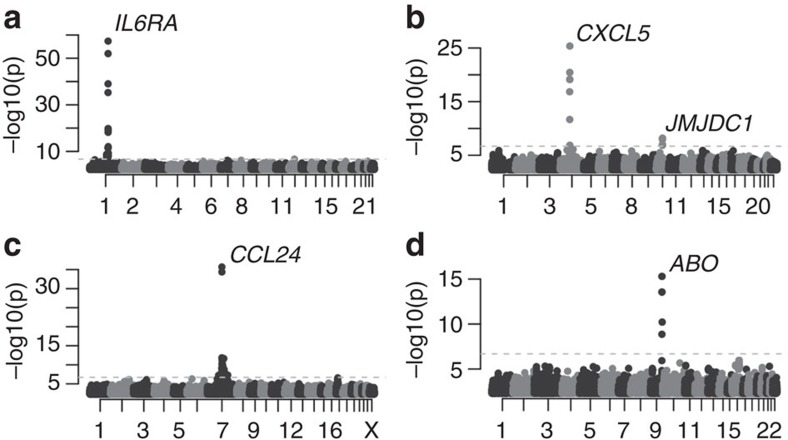
Manhattan plots of GWAS results. (**a**) IL-6RA (**b**) CXCL5 (**c**) CCL24 and (**d**) E-selectin. *X* axis labels refer to human chromosomes listed 1–22 and X. *P*-values were calculated from 1df Wald statistics *χ*^2^ values using 971 individuals.

All 970 individuals’ samples that passed the quality control (QC) were used to estimate the heritability for the 77 proteins with measurable levels by evaluating the co-segregation of the protein levels with the relatedness among individuals using a polygenic model (see Methods for details). In 75% (58 out of 77) of the proteins, the levels were found to be heritable (Bonferroni-adjusted *P*-value <0.05), with heritability ranging from 0.19 to 0.78 and the highest heritability for CCL24 (C-C motif chemokine 24; Supplementary Data 2). Thus, for a majority of the protein biomarkers, circulating levels are significantly affected by the individual’s genetic constitution. To determine the nature of the genetic effects on protein abundance, we performed association analyses using over 4.8M SNPs and INDELs identified by direct genotyping and whole-exome sequencing, followed by high-quality imputation. In this analysis, each of the 77 proteins was adjusted for the significant clinical and lifestyle variables (Table 1) and the samples were split into a discovery and a replication cohort based on sample collection round (see Methods for details). In the discovery phase, we identified 15 proteins with genome-wide significant hits (nominal *P*-value down to 1.1 × 10^−40^, Table 2), employing a Bonferroni-corrected *P*-value cutoff of 0.05. Of these, 14 had at least one replicated association (nominal *P*-value down to 1.1 × 10^−20^, Table 2). In all, 175 genome-wide significant hits were detected in the discovery phase, out of which 101 replicated. A combined analysis of all individuals revealed a total of 226 genome-wide significant hits in 14 proteins, with *P*-values down to 4.4 × 10^−58^, and a single marker explaining as much as 26.6% of the phenotypic variation seen after adjusting for the significant clinical and lifestyle factors (Table 2). A detailed description of each of the 226 hits, including overlaps with previous associations with any phenotype or trait, is given in Supplementary Data 3. IL-6RA (IL-6 receptor subunit alpha) showed the strongest association and the association was caused by one or very few SNPs located in the gene that encodes the respective protein, similar to the case for the majority of the biomarkers (Fig. 2a). Conditioning on the top-hit revealed that four of the proteins, CCL24, MIC-A (major histocompatibility complex class I polypeptide-related sequence A), CXCL5 and Ep-CAM (epithelial cell adhesion molecule), had hits independent of the highest-ranking SNP (Table 3). For CXCL5 (Fig. 2b) and Ep-CAM, the second SNP was located on a different chromosome, whereas for CCL24 (Fig. 2c) and MIC-A, the second SNPs were located close (<40 kb, Table 3) to the first hit. The second SNP for Ep-CAM explained 6.5% of the variance of the unadjusted phenotype, as compared with 4.9% for the top-ranking SNP. For the other three proteins, the fraction of variance explained by the second-ranking SNPs was small compared with the top-ranking SNP. For 12 of the 14 biomarkers with a strong genetic association (CCL24, CD40-L (CD40 ligand), CXCL5, CXCL10, Ep-CAM, IL-12B, IL-17RB (IL-17 receptor B), IL-6RA, hK11 (Kallikrein-11), MIA (melanoma-derived growth regulatory protein), MIC-A and VGEF-D), the top SNPs were located *in cis* with the gene encoding the protein. We compared our 226 hits with eQTLs as reported by the NCBI’s eQTL database and found overlapping SNPs in 11 cases. These were reported for IL-17RB (one SNP) and CCL24 (one SNP) in liver^23^ and for MIC-A (nine SNPs) in lymphoblastoids^24^ (Supplementary Data 3). As expression is cell type specific and eQTL studies only exist for a limited set of tissues, the number of SNPs found here to be eQTL is likely to be an underestimate. For two of the proteins (CCL19 and E-selectin), the genome-wide significant hits were located at other loci than the one coding for the protein (Table 3). The top hits for CCL19 were located in the major histocompatibility complex class II gene cluster, encoding molecules present on antigen-presenting cells and B-cell lymphocytes. CCL19 is a chemokine implicated in inflammatory and immunological responses, but also in normal lymphocyte recirculation and homing. Higher serum levels of CCL19 have been associated with poor prognostics of AIDS patients^25^. For E-selectin, the circulating level is known to be affected by ABO blood group. Here, even after correction for blood group at the A/B/0-level, the top hits in the GWAS were located within the *ABO*-gene, determining the blood group (Fig. 2d), with our top hit (rs507666) being a perfect tag SNP for the A1 subtype^26^, suggesting that the specification of the A group into A1 and A2 is involved. Our dependency of the E-selectin levels on ABO status is consistent with the pattern described earlier^15^, where individuals with the O blood type have the highest levels. This is in contrast to the patterns for TIE2 and PECAM-1, where individuals carrying the B or AB blood group have the highest values (Supplementary Fig. 1). For the other proteins (Ep-CAM, CCL19 and CXCL5), we found no evidence such as eQTLs or common pathways linking the loci that did not code the protein to the gene coding the protein. In summary, for a large number of the biomarkers, significant genetic effects on protein levels could be identified.

### Personalized biomarker-specific covariate profiles

**Figure 3:**
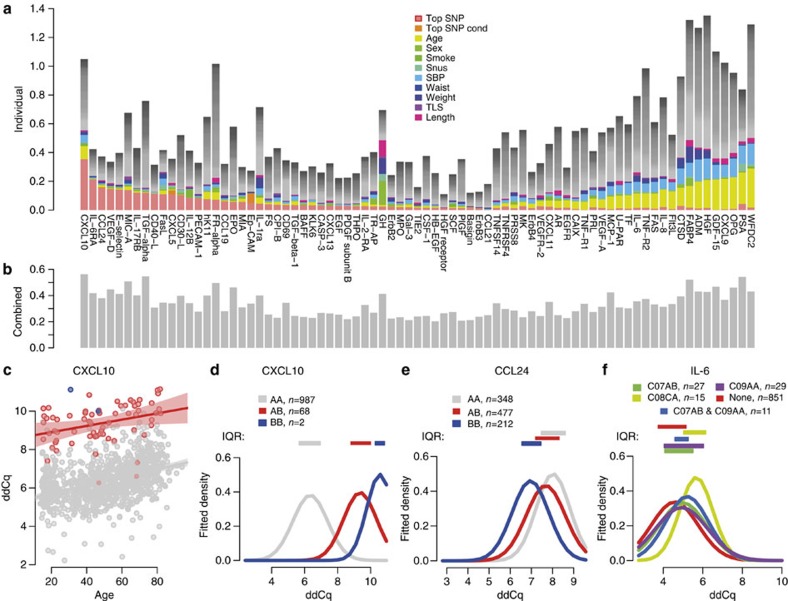
Covariates and protein biomarkers. (**a**) Variance explained by each of the covariates for the set of 77 biomarkers with measurable variability with the 11 most important covariates coloured. The combined effect of the remaining covariates is shown in grey, assuming independence in effect between covariates. (**b**) The percent of the variance explained by the full set of covariates studied for the 77 proteins, using a combined model. (**c**) Abundance of CXCL10, expressed as ddCq-values, in relation to age when stratified by genotype at rs11548618; AA (grey), AB (red) and BB (blue). Shadowed areas represent the 95% confidence interval in a linear model predicting ddCq from age. (**d**) Fitted normal distribution densities based on mean and standard deviation in ddCq-values for CXCL10, split by the rs11548618 genotype. (**e**) Fitted normal distribution densities based on mean and standard deviation in ddCq-values for CCL24 split by the rs6946822 genotype. (**f**) Fitted normal distribution densities based on mean and standard deviation in ddCq-values for IL-6 split by use of hypertension medications. Only groups where there are at least 10 individuals are shown. C07AB: β-blocking agents, selective. C08CA: dihydropyridine derivatives. C09AA: ACE inhibitors, plain. (**d**–**f**) Interquartile ranges indicated with coloured boxes above the curves.

The relative importance of individual genetic, clinical and lifestyle factors on the abundance differed dramatically between the 77 biomarkers (Fig. 3a). Some biomarkers were affected by strong genetic factors, whereas others mainly by environmental or clinical factors. These variables are not always independent, such as blood pressure and use of medication, which are both related to age. This can be seen in that the total fraction of observed variance, as determined by a combined model including all 158 covariates plus the top-ranking SNP and the top-ranking SNP from the conditional analysis, lie between 0.20 and 0.56 (Fig. 3b), whereas the sum of the explained variance by individual covariates in some cases reached above 1 (Fig. 3a). Figure 3 illustrates the main results of the study. Most of the 77 biomarkers showed large variation in abundance between individuals but they differed considerably with regard to the specific genetic, clinical or lifestyle factors involved. At one extreme, IL-6RA levels were affected most strongly by the individuals’ genotype and only a very small fraction of the variance was explained by other covariates, with BMI being the strongest (1.0%). Even assuming that all 158 covariates, besides the top ranking SNP, contributed independent effects, the sum of the fraction of the variance of IL-6RA levels explained by these factors was less (21.0%) than the single genetic effect (21.3%). At the other end of the spectrum, HGF did not show a significant heritability, and none of the genetic markers reached genome-wide significance. However, 17 other covariates were nominally significant (*P*-value<0.05) for HGF and 4 (weight, sample round, systolic blood pressure and age) remained significant after correction for multiple testing. These covariates accounted for 3.3%, 2.9%, 14.1% and 19.3%, respectively, of the percentage-of-variance-explained. In addition, the use of platelet aggregation inhibitors (ATC: B01AC) and loop-diuretics (ATC: C03CA) explained 5.7% and 7.1% of the variance observed in the unadjusted ddCq-values, respectively, whereas the top ranking SNP only accounted for 1.6%. In the middle part of the distribution in Fig. 3a, we find biomarkers that were less affected by the genetic, clinical or environmental factors studied, possibly reflecting limited non-disease-related variability.

Information on the set of important variables for each biomarker can be used to reduce the non-disease-related variation. For instance, soluble CXCL10, which shows elevated levels in patients with a number of autoimmune-related diseases^27^, has previously been shown to be associated with systolic blood pressure^28^. Here, we confirm the correlation with systolic blood pressure, which explains 5.6% or the variability, but we also found a significant correlation with age (9.0% of variability) and a very strong effect of genetic variants (35.4% of variability). Stratifying individuals on age did not appreciable reduce the range of variability (Fig. 3c). However, stratifying on the basis of the genotype at the top hit (rs11548618) had a considerable effect on reducing the variability (Fig. 3d). In the case of CCL24, the carriers of the reference allele of rs6946822 had a level 209% (linearized ddCq) of the average value of the homozygote carriers of the alternative allele (Fig. 3e). The effect of medication on the abundance can be demonstrated by IL-6 (Fig. 3f), where the distribution of protein level was clearly shifted upwards with the use of dihydropyridine derivatives (ATC: C08CA) found in drugs prescribed for treatment of hypertension or angina pectoris. Interestingly, this was the only hypertension medication that is correlated with higher IL-6 levels, and neither angiotensin-converting-enzyme (ACE)-inhibitors (ATC: C09AA), selective β-blocker agents (ATC: C07AB) nor a combination of these mediate this effect (Fig. 3f). This implies that detailed medication information may be needed for proper use of this biomarker.

### Availability

Full summary statistics of the combined results from the 14 GWAS’s with genome-wide significant hits are available from doi:10.5879/BILS/g000001.

## Discussion

We have shown that for 72 of the 77 biomarkers studied, the circulating plasma levels are strongly associated with genetic, clinical or lifestyle factors. Most biomarkers are highly heritable, and for 14 biomarkers, we identified strong genetic associations, with the top SNP explaining as much as 36% of the variability in protein abundance between individuals. For these biomarkers, stratifying patients based on their genotype may dramatically enhance the ability to detect deviations from normal circulating levels. A number of non-genetic factors also show a strong effect on biomarker levels, with age, systolic blood pressure and weight affecting a large number of the biomarkers. As cancer incidences increase with age^29^, so does the use of prescribed medications (Spearman’s rho, *R*^2^=0.29, Supplementary Fig. 2). Interestingly, we identified medication as an important clinical variable that should be considered when using the biomarkers for diagnosis or risk prediction. For instance, Basigin expression has been associated with shorter survival and proposed as a biomarker for adjuvant therapy in colorectal cancer^30^. Our analysis did not show any significant association of Basigin levels with covariates such as anthropometrics, age, sex or smoking. However, the use of glucocorticoids commonly found in inhalators used to treat asthma-related conditions, decreased circulating levels of Basigin thereby possibly masking the need for adjuvant treatment. Our results indicate that when using Basigin as a biomarker in an ageing population, medication history and dosage should be taken into account in order to establish an appropriate clinical cutoff. Another example is the IL-6 and IL-6 receptor (IL6-RA), where we confirm the strong effect of the genetic constitution on the circulating IL6-RA levels. We also show that medications used to treat, for example, hypertension such as dihydropyridine derivatives, but not ACE-inhibitors or selective β-blockers agents, cause or maintain an increase in the inflammatory response cascade via high IL-6 levels. The IL-6 signalling is important in the pathogenesis of several autoimmune and chronic inflammatory diseases^31^ and antibody-based drugs are used to target the IL-6 receptor in patients with RA in order to dampen the inflammatory response^32^. In clinical practice, only two-thirds of the patients treated with these drugs respond to the treatment and factors such as age and medical history have been shown to be predictors of remission and response in RA patients^33^. Future investigations are clearly needed to specifically address the long-term effects of commonly used medications in this perspective.

In a clinical context, circulating levels of CXCL10 have been estimated to 120±83 pg ml^−1^ in patients diagnosed with Graves’ Disease as compared with 72±32 pg ml^−1^ in controls^34^; an average increase of 67% not taking genetic and non-genetic covariates into account. By comparison, the average increase in individuals in our study carrying the reference genotype for rs11548618 was 178% (linearized ddCq) of the level in heterozygous individuals, clearly illustrating the relative importance of carrier genotype versus to disease state on biomarker levels. Previous efforts^35^,^36^ have identified genetic susceptibility loci for Graves’ disease but none of these overlap with the loci associated with the CXCL10 levels, suggesting that in this case the causal effects of the disease are not directly linked to the biomarker levels. Another strong genetic effect was observed for CCL24, where carriers of the reference allele of rs6946822 have a level 209% (linearized ddCq) of the average value of the homozygous carriers of the alternative allele (Fig. 3e). The worldwide minor allele frequency of rs6946822 is listed in NCBI's short genetic variation database (dbSNP) as 0.46, implying that every fifth individual will be homozygote, similar in frequency to the individuals who smoke in the United States today^37^, demonstrating the large, common genetic effects on biomarker variation found in the population today.

We also find biomarkers that are not significantly affected by any of the variables examined, rendering them less susceptible to variability induced by non-disease-related factors. Although we have investigated a large number of genetic, clinical and lifestyle factors, they altogether explain at most 56% of the variation in biomarker levels between individuals. The remaining variance must reflect other factors, or non-additive interaction between some of the factors studied, and their identification could further increase the utility of biomarkers by reducing sources of variation unrelated to disease state. For example, CCL24 had a heritability of 0.78, indicating that additional genetic loci might affect protein levels. For 15 of the biomarkers, the vast majority of abundances were below the detection limits in our cohort. Several of these could represent ideal biomarkers without major presence in normal plasma and thus with no influencing genetic or lifestyle factors. Among these was for instance mucin-16 (or CA125) that is used clinically as a test for ovarian cancer^38^ and also potential biomarkers such as regenerating islet-derived protein-4 that has been proposed as a biomarker for pancreatic ductal adenocarcinoma^39^.

This study identifies several previously unknown genetic and lifestyle factors influencing the circulating plasma levels of disease biomarkers in a population-based cohort, but has its limitations. First, we have a relatively small sample size (*N*=1005) for genetic association studies. Despite this fact, we identify and replicate 12 novel associations of large effect on the disease biomarkers. Although large GWAS consortia have identified hundreds of genetic variants associated with variation in disease-related phenotypes^40^,^41^, most of these SNPs are common and have such small effect sizes that they are not clinically useful.

Personalized cancer medicine is on a trajectory from long awaited promise to existing reality, with clinical applications for a small number of cancers with directed treatments. In chronic myelogenous leukemia, patients with a specific translocation respond well to treatment with a tyrosine-kinase inhibitor blocking an enzyme that in turns triggers signalling cascades^42^. Also, patients with non-small-cell lung cancer and a gene-fusion mutation have higher drug response rates than those lacking this gene fusion^43^. However, the number of cancer biomarkers in clinical use is still limited. In the set of biomarkers studied here, we identified a surprisingly strong genetic effect on some biomarkers after correcting for clinical (medication) and lifestyle variables. Likewise, other biomarkers were strongly affected by environmental lifestyle or clinical factors. Genotyping of selected polymorphisms with a strong effect on abundance appears to be crucial for about 20% of the biomarkers in our study, whereas lifestyle and medication are important covariates for the majority. In the daily clinical routine, we envision that analysis of broad-spectrum biomarkers could be used as a follow-up analysis for patients, or for screening of risk groups. Our analysis indicate that such tests would be accompanied by collecting additional relevant information such as anthropometrics, medication and genotyping of specific polymorphisms known to affect the baseline of these biomarkers. The clinical laboratory that performs the biomarker analysis would have documentation on which cofactors that significantly influence the baseline levels, and could advise the physician on how to interpret the outcome of the test. Our results imply that using biomarker-specific covariate profiles will make it possible to determine more precise, individualized, clinical cutoff levels. This in term could lead to a more efficient use of protein biomarkers for early detection of abnormal levels and for increased sensitivity and specificity in disease diagnosis. By employing biomarker-specific profiles of covariates it will be possible to fully harness the potential of existing and novel biomarkers for disease diagnosis and management.

## Methods

### Samples

The NSPHS was initiated in 2006 to provide a health survey of the population in the parish of Karesuando, county of Norrbotten, Sweden, and to study the medical consequences of lifestyle and genetics. This parish has about 1,500 inhabitants who meet the eligibility criteria in terms of age (≥15 years), of which 719 individuals participated in the study (KA06 cohort). As a second phase of the NSPHS, another 350 individuals from a neighbouring village (Soppero) were recruited in 2009 (KA09 cohort). For each participant in the NSPHS, blood samples were taken (serum and plasma) and stored at −70 °C on site. Both the 2006 and 2009 samples used in this study have undergone two freeze–thaw cycles before the measurements carried out here. DNA has been extracted for genetic analyses and detailed descriptions of this study have been published elsewhere^44^,^45^,^46^. A questionnaire was used to collect data on medications and lifestyle. The questionnaire was filled in at the local health-care centre in the presence of the local district nurse. Notably, around 15% of the participants of the study adhere to a traditional lifestyle based on reindeer heading and crafts. Differences in, for example, diet in this group compared with the group with a lifestyle typical of more industrialized regions have been shown to increase levels of circulating blood lipids^47^, which motivates to include the traditional lifestyle adherence as a covariate.

### Ethical considerations

The NSPHS study was approved by the local ethics committee at the University of Uppsala (Regionala Etikprövningsnämnden, Uppsala, 2005:325) in compliance with the Declaration of Helsinki^48^. All participants gave their written informed consent to the study including the examination of environmental and genetic causes of disease. In cases where the participant was not of age, a legal guardian signed additionally. The procedure that was used to obtain informed consent and the respective informed consent form has recently been discussed in light of present ethical guidelines^49^.

### Multiplexed PEA

Protein levels in plasma were analysed using the Olink Proseek Multiplex Oncology I ^96 × 96^ kit and quantified by real-time PCR using the Fluidigm BioMark HD real-time PCR platform as described earlier^10^. In brief, for each measured protein, a pair of oligonucleotide-labelled antibodies probes bind to the targeted protein, and if the two probes are in close proximity, a PCR target sequence is formed by a proximity-dependent DNA polymerization event and the resulting sequence is subsequently detected and quantified using standard real-time PCR. Each plate contains 96 wells whereof 92 are samples, 1 is a negative control and 3 are positive controls (spiked in IL-6, IL-8 and VEGF-A). Each sample is also spiked in with two incubation controls (green fluorescent protein and phycoerythrin), one extension control and one detection control. These controls are used to determine the lower detection limit (negative control) and to normalize the measurements into ddCq values according to the following formulae


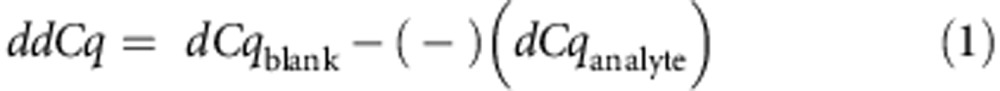


where


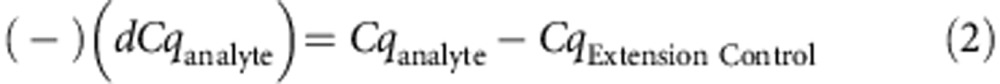


and 
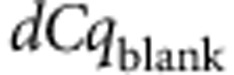
 is a per-assay value defined by the manufacturer to give a positive log2-scale. The ddCq values were then log2-transformed for subsequent analysis. Each PEA measurement has a specified lower detection limit calculated based on negative controls that are included in each run and measurements below this limit were removed from further analysis. Individual samples where at least one of the internal controls contained an outlier value (*n*=35) or where too many (>75%) measurements were below detection limits in any PEA (*n*=1) were also excluded from further analyses (total *n*=35 of 1005, 3.5%). We wanted at least 200 observations per protein above detection limit in order to conduct the downstream statistical analyses and therefore proteins with fewer observations were excluded from further analyses. After individual and protein quality control, 77 proteins measured in 970 individuals remained. Out of the removed proteins, seven proteins (betacellulin, epiregulin, IL-2, CA242, estrogen receptor, G-CSF and stromelysin-1) had 100% of measurements below detection limit. Uniprot recommended short names have been used throughout when these are available otherwise; the assay manufacturers’ abbreviations have been used. All assay characteristics including detection limits and measurements of assay performance and validations are available from the manufacturer’s webpage (http://www.olink.com/products/proseek-multiplex/downloads/data-packages).

### Genotype data

The KA06 and KA09 cohorts have previously been genotyped on the Illumina Infinium HapMap300v2 BeadChip (308,531 markers; Illumina) and Illumina Human OmniExpress BeadChip (731,442 markers; Illumina) arrays, respectively, as described earlier^8^. In brief, the specific KA06 and KA09 data were quality checked separately leaving 691 individuals with 306,086 SNPs at 99.50% genotyping rate and 346 individuals 631,503 SNPs at 99.88% genotyping rate, respectively. Four individuals were present in both cohorts and these were removed from the KA06 data. Here, we also genotyped the individuals from both cohorts (*n*=1059) on the Illumina Human Exome Beadchip containing 247,901 SNPs, insertions and deletions primarily selected to have coding changes. The genotype calling was done with the software GenomeStudio 2011.1 (Illumina Inc.) using a Project Sample generated Cluster File as recommended by the manufacturer. The Exomechip data were quality controlled requiring 95% and 98% genotyping rate on marker and individual levels, respectively, and a Bonferroni-corrected Hardy–Weinberg cutoff of 0.05 leaving 242,519 markers at a total genotyping rate of 99.94% in the 1,033 unique individuals previously genotyped. This analysis was carried out using custom R-scripts and PLINK (v1.07)^50^.

### Exome sequencing

We selected 100 individuals, 68 from KA06 and 32 from KA09, for Whole-Exome Sequencing using Agilent’s SureSelect system (Agilent) for exome capture and the SOLiD 5500xl instrumentation for sequencing. Each sample was sequenced to at least 30X coverage. The individuals were selected to represent as much genetic variation of the cohort as possible^51^. Alignment was done using the LifeScope software, and SNPs and INDELs were called using diBayes. For each position (*n*>1.5 M) where any individual so far sequenced at the Uppsala Genome Centre had called SNP or INDEL, we then checked our 100 individuals for coverage in order to differentiate between missing and reference calls. These positions were included to maximize the overlap with the 1,000 genomes reference panels to ensure proper imputation using two reference panels. Reference calls for SNPs were made if there were at least three reference sequence reads with unique start points and a maximum of 5% reads with non-reference at that position. Reference calls for INDELs were made if there was no reads at all without the reference call. All other calls were set to missing. We then required at most 5% missing call rate per SNP or INDEL. This resulted in 83,568 SNPs with non-zero MAF at 98.74% total genotyping rate and 38,290 INDELs with a total genotyping rate at 99.45% and an additional 350k positions with reference calls only. We then required a genotyping rate of 95% in both individual and marker level and a Bonferroni-corrected Hardy–Weinberg cutoff at 0.05, which resulted in 468,630 markers at total genotyping rate of 98.79%.

### Imputation of genotype data

We created an in-house reference panel to be used simultaneously with the 1,000 genomes^52^ reference panel^53^. The in-house panel was based on the 100 exomed individuals by merging the SNPs and INDELs called from the exomes with the SNPs common between the Illumina Human HapMap300v2 (used in the KA06 cohort) and the Illumina Human OmniExpress (used in KA09 cohort), *n*=182,916, and all the markers from the Illumina Human Exome chip. In this step, there was no additional filtering done on minor allele frequency in order to maximize the overlap with the SNPs in the 1,000 genome panel. The total number of markers in the in-house reference panel was 847,855. The reference haplotypes were created using in-house R-scripts, PLINK (v1.07) and phased using SHAPEIT (v2.r)^54^. Data were then imputed for the two cohorts separately using IMPUTE2 (v2.3.0) with a pre-phasing approach^55^. The input data were phased chromosome-wise using SHAPEIT (v2.r). In addition to our in-house panel, we also utilized the 1,000 Genomes Phase I integrated variant set (National Center for Biotechnology Information build b37, March 2012) accessed from the IMPUTE Web resource^53^. IMPUTE2 was run with the default parameters with the following changes ‘-- merge-ref-panels’ and ‘-k_hap 500 200’. The latter instructing IMPUTE2 to use 500 haplotypes from the 1,000G reference panel and all 200 from our in-house panel. Data were imputed in chunks of around 5M bases ensuring at least 200 genotyped SNPs in each chunk. No chunks spanned across the centromeres. The para-autosomal and non-para-autosomal regions on chromosome X were handled separately. The resulting data were filtered on marker level by requiring IMPUTE’s ‘info’ score >0.3 in both the KA06 and KA09 cohorts before merging. Merging of the imputed data was done using GTOOL (v0.7.5)^56^ requiring a dosage threshold above 0.9 in at least 95% of the individuals. The resulting merged data were further filtered using QCTOOL (v1.3)^57^ requiring a Bonferroni-corrected Hardy–Weinberg cutoff of 0.05 and a minor-allele frequency corresponding to at least one chromosome in the whole material. The final data set included 4,840,842 SNPs and INDELs.

### ABO blood group assignment

We assigned blood groups according to the ABO-system to our samples based on their genetic status of four genotyped SNPs (rs505922, rs8176746, rs8176704 and rs574347) in the region of the *ABO* gene. These four SNPs allow for accurate assignment of both the A/B/O groups and subtyping of A into A1 and A2 and subtyping of O into O01 and O02 (ref. 58). Using this approach, we successfully assigned blood groups to 97.9% of our samples.

### Statistic analyses

All statistical analysis was conducted in R^59^ and illustrations were produced using R and the Circos software^60^. Correlation between proteins and relevant variables was calculated separately for each measured protein by fitting a generalized linear model using the ’glm’ function including all covariates simultaneously. The significance of the each covariate’s contribution to the total variance was estimated using an analysis of variance approach as implemented by the ‘anova.glm’ function on the resulting generalized linear model. Covariates were considered significant for a specific protein if their Bonferroni-adjusted *P*-values were below 0.05 (*P*-value<3.16 × 10^−4^, 0.05/158). Each PEA measurement was individually adjusted for significant covariates (Supplementary Data 2) and rank-transformed to normality by using the ‘rntransform’ function available from the R-package GenABEL (v1.6.7)^61^. Correlations between pairs of PEA measurements were carried out, on the adjusted and rank-transformed values, using the ‘cor’ function applying Spearman’s Rho statistics on pairwise complete observations.

The NSPHS is a population-based study and includes many relatives and special care has to be attributed to avoid relational biases. Therefore, all genetic association calculations were carried out using the GenABEL or ProbABEL^61^ software suites, which has been developed to enable statistical analyses of genetic data of related individuals. These packages includes functions for estimating the narrow-sense heritability (*h*^2^) and performing genetic association analyses^62^ by adjusting for pedigree structure. In brief, the heritability of each trait (protein abundance) is estimated using a polygenic model as implemented by the ‘polygenic’ method in the GenABEL R-package^61^. This heritability estimate represents the variance in the phenotype that is explained by genetic factors and is estimated by maximizing the likelihood of the trait-data under a polygenic model including fixed effects such as covariates and relatedness among individuals (kinship). The result of the ‘polygenic’-call contains the inverse variance–covariance matrix of the estimates and trait residuals and is included in the downstream association calculations together with the posterior genotypic probabilities. Specifically, these calculations are performed using the ProbABEL programme using the ‘--mmscore’ option. Kinship matrix calculations were carried out using the autosomal markers shared (*n*=182,916) between the two types of genotyping arrays used in the KA06 and KA09 cohorts. Contribution of single SNP’s to phenotypic variation on the unadjusted ddCq values was calculated in R by fitting a linear model (using ‘lm’) with ddCq values as response and the posterior genotypic probabilities as terms and fraction of variance explained was determined from the resulting model using ‘summary.lm’. Fraction of variance explained by a single SNP in the adjusted phenotypes including effects of relatedness was estimated by dividing the resulting chi-square test score (from ProbABEL) with the number of samples used.

The KA06 cohort was used as discovery cohort in the GWAS and KA09 as replication cohort. As we cannot rule out protein degradation effects due to differences in storage time between the two cohorts, this split is favourable to a random split where degradation effects could affect the association analysis. Strict Bonferroni-adjusted *P*-values (*P*-value<1.03 × 10^−8^, 0.05/4,840,842) were used to report significance in the discovery cohort and the replication cohort (*P*-value<0.05/number of significant SNPs in the discovery cohort). We also ran a combined analysis with the same cutoff used as in the discovery phase. For all proteins with replicated hits, a conditional analysis was carried out in which the genetic associations were re-calculated using the dosage values of the top-ranking SNP as covariate. This analysis was only run in the combined material and on chromosomes that had hits in that replicated in the discovery-replication phase and *P*-value <5 × 10^−8^ was used as cutoff.

## Author contributions

S.E. designed experiments, developed analysis tools, analysed data and wrote the paper. Å.J. contributed to the design of the experiments. S.B.E. interpreted results. U.G. conceived of the study, designed the experiments and wrote the paper.

## Additional information

**How to cite this article:** Enroth, S. *et al*. Strong effects of genetic and lifestyle factors on biomarker variation and use of personalized cutoffs. *Nat. Commun.* 5:4684 doi: 10.1038/ncomms5684 (2014).

## Supplementary Material

Supplementary Figures Supplementary Figures 1-2

Supplementary Data 1 Information on the biomarkers.

Supplementary Data 2 Information on which covariates that significantly influence the individual proteins.

Supplementary Data 3 Detailed information on the hits from the GWAS.

## Figures and Tables

**Table 1:** List of significant covariates.

**Covariate**	**No. of proteins**	**Protein**
Age	24	Up: ADM, CTSD, CXCL9, CXCL10, CXCL11, ErbB4, FAS, Flt3L, GDF-15, WFDC2, HGF, IL-8, IL-2-RA, KLK6, hK11, MCP-1, OPG, PSA, TF, TNF-R1, TNF-R2 U-PAR, VEGF-ADown: E-selectin
Sex (female)	4	Up: GH, FABP4Down: E-selectin, TR-AP
Systolic blood pressure	22	Up: ADM, CTSD CXCL9, CXCL10, ErbB4, FAS, Flt3L, hK11, GDF-15, WFDC2, HGF, IL-8, TGF-beta-1, MCP-1, OPG, PRSS8, PSA, TF, TNF-R1, TNF-R2, U-PAR, VEGF-A
Length	11	Down: ADM, BAFF, CXCL9, FABP4, Flt3L, FR-alpha, GDF-15, GH, WFDC2, OPG, U-PAR
Weight	21	Up: ADM, CTSD, CSF-1, CXCL10, CPI-B, E-selectin, ErbB2, FABP4, FAS, GDF-15, HGF, IL-1ra, IL-6, MCP-1, OPG, PRSS8, TNF-R1, TNF-R2, U-PAR, VEGF-ADown: GH
Waist	3	Up: GDF-15, WFDC2, OPG
TLS (147 ppl)	4	Down: FABP4, GH IL-6, IL-2RA
KA06 (720 ppl)	17	Up: CCL21, HGF, MCP-1, MK, PECAM-1Down: CASP-3, CD69, CXCL11, EGF, IL-1ra, IL-2-RA, hK11, MIC-A, OPG, TNFSF14, TNF-R1, TNF-R2
Smoking (130 ppl)	2	Up: WFDC2Down: IL-12
A10AD (3 ppl)	1	Up: TGF-alpha
C03DA (2 ppl)	4	Up: CXCL13, MIC-A, U-PAR, TNF-R2
C08CA (54 ppl)	1	Up: IL-6
N05CD (2 ppl)	1	Up: PRL
N05CF (6 ppl)	2	Up: FAS, IL-1ra
R03BA (26 ppl)	2	Down: Basigin, HGF-receptor
R03AC (13 ppl)	1	Down: VEGF-D
R03DC (3 ppl)	1	Down: VEGF-D
ABO blood group	3	E-selectin, PECAM-1, TIE2

TLS, traditional lifestyle.

Direction of correlations was calculated using PEA-values without any additional covariate correction having been carried out. A10AD (insulins and analogues for injection, intermediate-acting combined with fast-acting), C03DA (aldosterone antagonists), C08CA (dihydropyridine derivatives), N05CD (benzodiazepine derivatives), N05CF (benzodiazepine-related drugs), R03BA (glucocorticoids), R03AC (selective β-2-adrenoreceptor agonists), R03DC (leukotriene receptor antagonists).

**Table 2:** GWAS results.

	**Discovery phase**					**Replication phase**					**Combined phase**				
**Protein**	H*	***N***	**SNPs**	***P*** **(best)**	**% Var Expl**†	H*	***N***	**SNPs**	***P*** **(best)**	**% Var Expl**†	H*	**SNPs**	***P*** **(best)**	**% Var Expl**†	***λ***‡
IL-6RA	0.66	653	22	1.1 × 10^−40^	27.3	0.50	317	10	1.1 × 10^−20^	27.4	0.68	22	4.4 × 10^−58^	26.6	1.06
CXCL10	0.47	641	24	1.4 × 10^−31^	21.3	0.39	311	1	2.3 × 10^−06^	7.2	0.46	16	6.8 × 10^−37^	16.9	0.94
CCL24	0.71	653	17	2.3 × 10^−28^	18.7	0.79	317	4	5.3 × 10^−11^	13.6	0.78	30	2.0 × 10^−36^	16.4	1.09
MIC-A	0.40	363	25	2.1 × 10^−17^	19.8	0.54	174	14	3.7 × 10^−04^	7.3	0.52	19	5.3 × 10^−16^	12.2	1.25
CD40-L	0.27	640	14	1.3 × 10^−16^	10.7	0.39	315	13	2.1 × 10^−10^	12.8	0.33	22	1.1 × 10^−25^	11.5	1.13
CXCL5	0.41	653	8	7.7 × 10^−16^	9.9	0.62	317	5	1.5 × 10^−10^	12.9	0.51	8	4.3 × 10^−26^	11.5	1.06
hK11	0.33	628	5	3.7 × 10^−15^	9.8	0.08	310	5	4.3 × 10^−04^	3.4	0.24	15	5.3 × 10^−18^	7.9	0.98
Ep-CAM	0.54	653	18	2.1 × 10^−14^	5.2	0.50	317	8	1.4 × 10^−09^	11.6	0.57	24	6.7 × 10^−16^	6.7	0.97
IL-17RB	0.51	415	1	4.7 × 10^−13^	12.6	0.30	199	1	1.1 × 10^−07^	14.2	0.48	2	1.7 × 10^−18^	12.5	0.96
IL-12B	0.38	652	1	4.7 × 10^−12^	7.3	0.64	317	1	1.3 × 10^−06^	7.4	0.43	4	9.0 × 10^−17^	7.1	0.93
VEGF-D	0.29	652	21	8.7 × 10^−10^	5.8	0.39	317	21	6.9 × 10^−08^	8.8	0.36	36	1.1 × 10^−15^	6.6	1.01
E-selectin	0.44	639	4	8.9 × 10^−10^	5.7	0.70	306	4	1.3 × 10^−08^	10.6	0.56	6	5.0 × 10^−16^	7.0	1.01
MIA	0.25	648	4	1.6 × 10^−09^	5.6	0.55	316	4	2.0 × 10^−09^	11.4	0.37	7	6.8 × 10^−17^	7.2	1.05
MPO	0.43	646	1	3.8 × 10^−09^		0.22	317	0	9.0 × 10^−01^		0.39	0	6.1 × 10^−07^		1.05
CCL19	0.32	653	10	1.3 × 10^−08^	5.0	0.33	317	10	2.0 × 10^−06^	7.1	0.32	15	2.6 × 10^−13^	5.5	0.92

GWAS, genome-wide association study; SNP, single-nulecotide polymorphism; Var Expl, variance explained.

*P*-values were calculated from 1df Wald statistics *χ*^2^-values.

^*^Heritability estimate.

^†^Fraction of variance explained in the adjusted and transformed phenotype by the top-ranking SNP (SNP with lowest *P*-value in the combined analysis).

^‡^Estimation of the inflation factor for the resulting distribution of *P*-values.

**Table 3:** Location and annotation of top GWAS hits.

**Protein**	**SNP**	***P*****-value**	**Effect,** ***β*** **(s.e.)**	**Effect allele (reference)**	chr:position*	**Gene**	**Type**
IL-6RA	rs4129267	4.39 × 10^−58^	0.84 (0.052)	T (C)	1:154426264	*IL6RA*	Intronic
CXCL10	rs11548618	6.78 × 10^−37^	1.80 (0.14)	A (G)	4:76943947	*CXCL10*	Nonsynonymous
CCL24	rs6946822	2.02 × 10^−36^	−0.62 (0.049)	T (C)	7:75479448	*CCL24*	Intergenic, 36 kb upstream
	rs11465293†‡	7.95 × 10^−13^	−0.63 (0.088)	A (G)	7:75442723	*CCL24*	Nonsynonymous
MIC-A	rs3869132	5.33 × 10^−16^	−0.73 (0.090)	A (G)	6:31410948	*MIC-A*	Intergenic, 28 kb downstream
	rs2263316†	1.02 × 10^−08^	0.48 (0.083)	G (A)	6:31421297	*MIC-A*	Intergenic, 38 kb downstream
CD40-L	rs148594123	1.07 × 10^−25^	−0.96 (0.091)	A (G)	X:135741443	*CD40LG*	Nonsynonymous
CXCL5	rs425535§	4.27 × 10^−26^	−0.86 (0.081)	T (T)	4:74863997	*CXCL5*	Synonymous
	rs2472649||	3.57 × 10^−21^	−0.70 (0.074)	A (A)	4:74857708	*CXCL5*	Intergenic, 4 kb downstream
	rs2393967†	4.54 × 10^−08^	0.30 (0.052)	C (A)	10:65133156	*JMJD1C*	Intronic
hK11	rs117268623	5.25 × 10^−18^	−1.48 (0.17)	T (C)	19:51527970	*KLK11*	Nonsynonymous
Ep-CAM	rs201314303§	6.74 × 10^−16^	−2.48 (0.31)	G (C)	2:47612302	*EPCAM*	Intronic
	rs56398830†	1.26 × 10^−15^	−0.94 (0.12)	A (G)	13:103701690	*SLC10A2*	Nonsynonymous
IL-17RB	rs6801605	1.75 × 10^−18^	−0.56 (0.064)	A (G)	3:53876218	*CHDH*	Intronic
						*IL17RB*	4 kb upstream
IL-12	rs10045431	8.99 × 10^−17^	0.47 (0.057)	A (A)	5:158814533	*IL12B*	Intergenic, 57 kb upstream
VEGF-D	rs188779336§	1.11 × 10^−15^	−1.58 (0.20)	G (C)	X:15308292	*ASB11*	Intronic
	rs146086561¶	1.81 × 10^−15^	−1.57 (0.20)	T (C)	X:15365438	*FIGF*	Nonsynonymous
E-selectin	rs507666	5.01 × 10^−16^	−0.55 (0.067)	A (G)	9:136149399	*ABO*	Intronic
MIA	rs2230694§	6.83 × 10^−17^	0.66 (0.079)	G (A)	19:41263403	*SNRPA*	Synonymous
	rs2607426||	1.13 × 10^−16^	0.65 (0.079)	G (A)	19:41274713	*MIA*	Intergenic, 6k upstream
	rs2233154¶^,^§	1.13 × 10^−16^	0.65 (0.079)	T (C)	19:41281346	*MIA*	UTR5
	rs2233159¶^,^§	1.07 × 10^−16^	−0.65 (0.079)	C (C)	19:41283365	*MIA*	UTR3
CCL19	rs7775228	2.63 × 10^−13^	0.51 (0.069)	C (T)	6:32658079	*HLA-DQB1*	Intergenic, 24 kb upstream

GWAS, genome-wide association study; SNP, single-nulecotide polymorphism.

*P*-values were calculated from 1df Wald statistics *χ*^2^-values.

^*^In hg19 coordinates.

^†^Independent genome-wide significant loci as per conditional analysis on top-snp, reported *P*-values after conditioning on the top-hit.

^‡^rs11465293 was not discovered in the unconditional discovery-replication analysis and was subsequently rerun in a conditional discovery-replication analysis resulting in the *P*-values 3.9 × 10^−9^ and 6.7 × 10^−5^ for the discovery and replication cohorts, respectively.

^§^Imputed.

^||^When the top SNP(s) was imputed, the top ranking genotyped SNP was also included in the table.

^¶^In perfect LD (*R*^2^=1) with top-snp in our cohort.
