# Supplementary material for: Strong effects of genetic and lifestyle factors on biomarker variation and use of personalized cutoffs
Source: Nat Commun. 2014 Aug 22;5:4684. doi: 10.1038/ncomms5684 (PMC4143927; doi:10.1038/ncomms5684)
Supplement: Supplementary Figures — 1-2 [file ncomms5684-s1.pdf]

Supplementary Figures

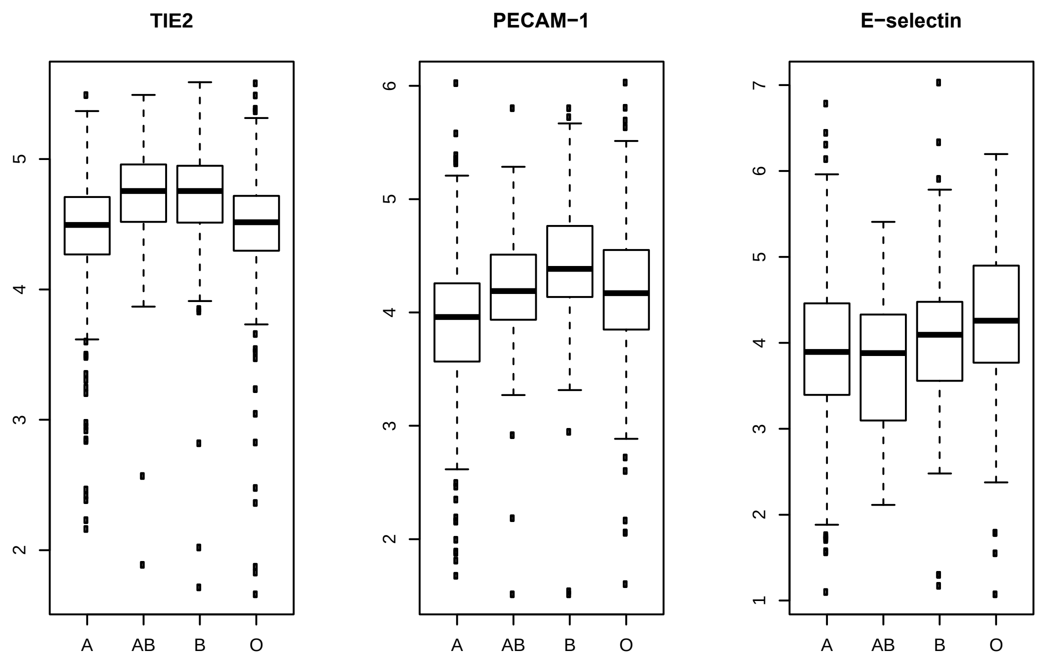

Supplementary Figure 1. Distribution of raw ddCq values based on blood group. Y-axes are raw ddCq values of protein level abundances split on A/AB/B and O blood group assignment. Thick horizontal lines depicts the median, the boxes depicts the first and third quartiles.

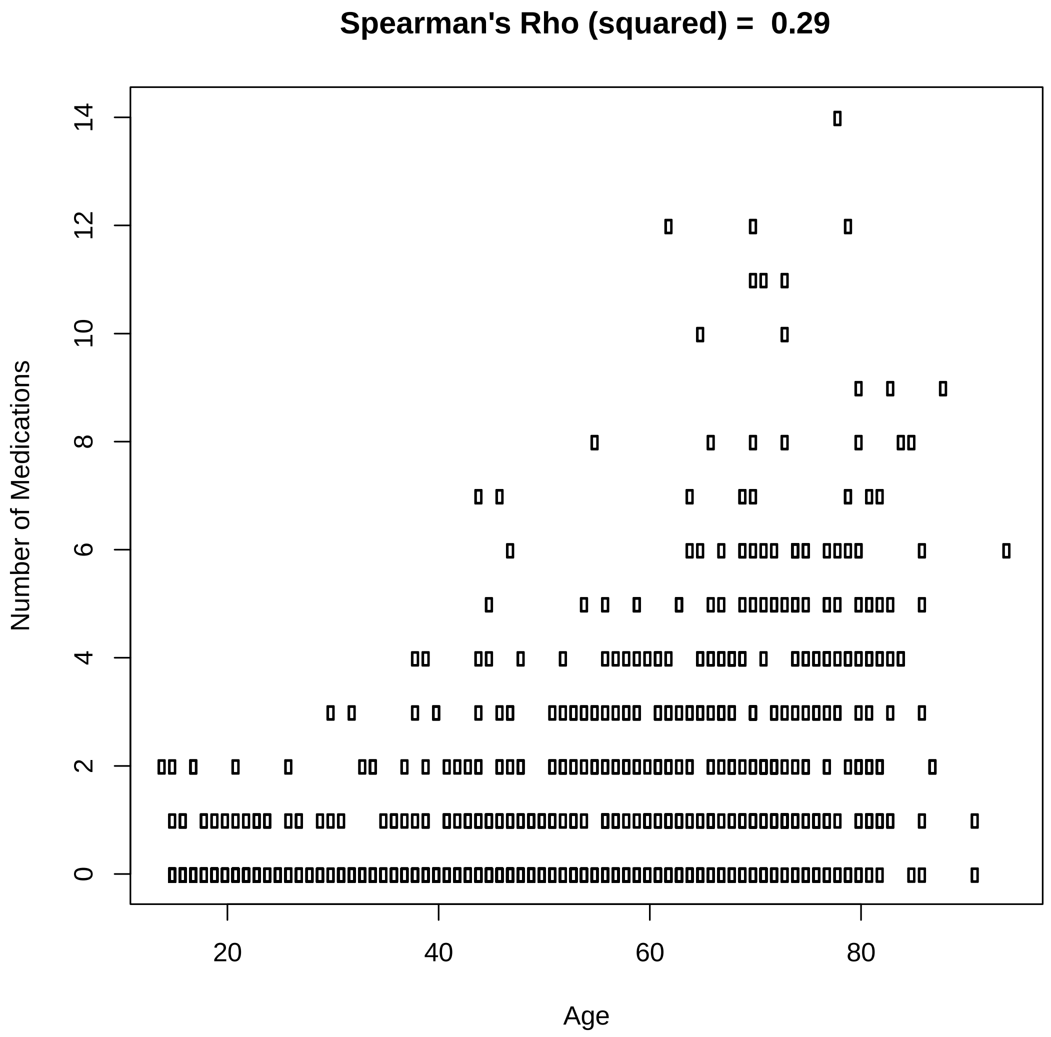

Supplementary Figure 2. Correlation between age and number of medications listed. Y-axis denotes the number of medications an individual is currently taking and the X-axis the age of the individuals.
